# Classification–coordination–collaboration: a systems approach for advancing Sustainable Development Goals

**DOI:** 10.1093/nsr/nwaa048

**Published:** 2020-03-19

**Authors:** Bojie Fu, Junze Zhang, Shuai Wang, Wenwu Zhao

**Affiliations:** 1 State Key Laboratory of Earth Surface Processes and Resource Ecology, Faculty of Geographical Science, Beijing Normal University, China; 2 State Key Laboratory of Urban and Regional Ecology, Research Center for Eco-Environmental Sciences, Chinese Academy of Sciences, China

## Abstract

It is an urgent task to advance Sustainable Development Goals (SDGs) on different scales in the world. We propose a systems approach to combat this issue, namely `classification–coordination–collaboration'. This approach allows SDGs to realize key breakthroughs over the short-term while achieving sweeping progress over the long run.

The SDGs provide a blueprint for the world's sustainable-development plan for 15 years from 2016 to 2030 [1]. However, recent reports suggest that we may not have enough time to achieve this vision [2,3]. Furthermore, both the complexity of relationships between the SDGs and differences in resources, development capabilities, needs and cultural features exacerbate our ability to achieve these goals [3,4].

Faced with these challenges, scientists have explored strategies to accelerate the implementation of SDGs from multiple perspectives, including analysing the interlinkages between SDGs [4], prioritizing the goals [5] and analysing the essential transformations [6]. However, due to the scale effect, a single country's program may have negative externalities for other countries [3]. To avoid this risk, a comprehensive and systematic framework is needed to promote joint action by countries, thus enabling SDGs to advance on a regional, national and global scale.

Based on the summary of relevant studies and actions over recent years, we propose a systems approach, namely classification–coordination–col-laboration (3C), to promote the realization of SDGs. The 3C approach provides a feasible way in which to promote the overall implementation of SDGs in various countries, consisting of the necessary processes and essential means for advancing SDGs (Fig. 1).

**Figure 1.**
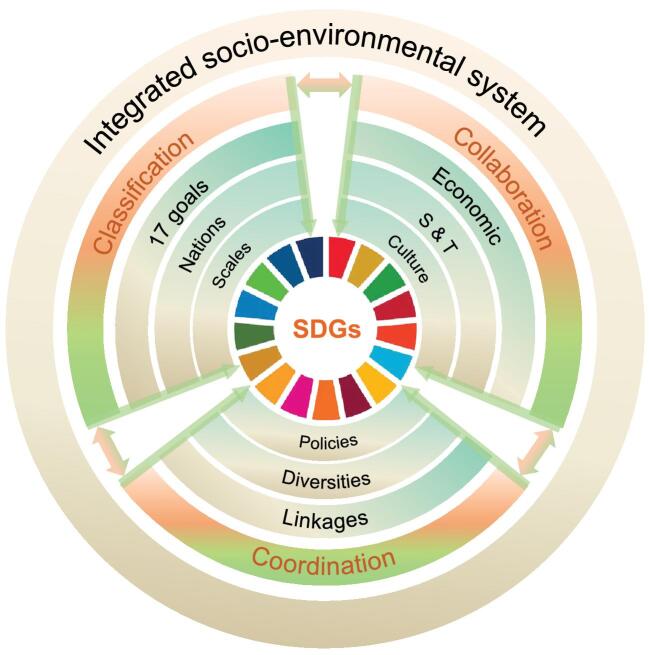
A systems approach for advancing the SDGs: classification–coordination–collaboration. Among the 3C constituents, classification is the foundation, which aims to identify the main features of the different SDGs, the differences among countries and the influence of scale; coordination is the core link, which aims to ensure policy coherence among countries and departments by formulating reasonable policies; and collaboration is the necessary means to achieve the SDGs as a whole. Our approach calls for significantly greater collaboration in economic, S&T and cultural fields, being consistent with the purpose of the four levers [3], which can accelerate the transformations to achieve the SDGs. In this approach, each constituent interacts with one another. For example, classification requires the coordination and collaboration of multi-stakeholders, and classification can also enhance coordination and promote more efficient collaboration.

## CLASSIFICATION

Classification refers to the assignment of objects to groups by considering their properties, grades or other characteristics. This process can lay the foundation for relational analysis, comparison and the joint management of objects. The 2030 Agenda states that all 17 SDGs are integral and indivisible [1]. Nevertheless, it is more than just a simple binding of individual SDGs, but instead requires a systems approach in which each goal is interrelated. Hence, we need to cluster the goals from a systematic perspective to achieve the maximum benefits with the minimum investment through appropriate governance measures [7].

In addition, differences in needs and capacities from which to respond to challenges in countries are also a key factor limiting the realization of SDGs [5]. It is necessary to clarify and classify the advantages and disadvantages of different countries, such as the official national classification system of the United Nations (https://unstats.un.org/sdgs/indicators/database/). These categories can be used as a reference, based on assessing the progress and further analysing the restrictive factors of different countries (that can then be clustered), to lay a foundation for promoting joint action.

Finally, we must consider changes on a space-time scale. The SDGs were proposed on a global scale and are not necessarily suitable for smaller scales; thus, it is imperative to pay attention to the representativeness and the comparability of indicators in the process of localizing SDGs. In terms of time, SDGs make up a long-term plan and it would be futile to attempt to achieve all goals simultaneously [3]. Consequently, we must clarify the prioritization of the goals over different periods to ensure that we can realize short-term breakthroughs while achieving comprehensive progress in the long term.

## COORDINATION

Coordination is a top-down management process. Modern management experts believe that coordination is the essence of management [8]. In SDGs-oriented management, coordination is the core link, as the purpose of coordination is not only to integrate the various components, but also to synchronize the functions of the various departments in order to achieve the SDGs with minimal effort. In doing so, we first should strengthen the coordination between the SDGs. This means that we must analyse the interactive mechanisms between each goal based on the classification discussed above and should involve a consensus of the multidisciplinary scientific community.

Second, we need to be mindful of the differences in capabilities and needs among different countries [5]. Currently developed countries, as well as some developing countries, have led economic development and technological innovation, while lesser-developed countries have yielded higher scores in natural-resource protection and tackling climate change—the likes of which are a partial result of lessened gross natural-resource exploitation and extraction. Therefore, by coordinating the strengths and weaknesses of different countries under the auspices of the UN initiatives, the implementation of the SDGs can be accelerated.

Third, we must pay attention to the coordination of cross-scale management policies. Central, regional and local governments should take actions based on the results of the linkages between the SDGs from the scientific community. Policies that have repetitive impacts on certain goals should be integrated (or repealed) to reduce management costs and appropriate policies should be implemented to make up for the deficiencies. Finally, as no country can concurrently cope with all the SDGs, governments should take actions to ensure consistency between short- and long-term policies.

## COLLABORATION

Collaboration is having multiple individuals or organizations working together to accomplish a task [9]. Compared to coordination, collaboration is a bottom-up, as well as a top-down, process; it can spontaneously occur through the initiatives of different individuals or organizations. The collaboration among different stakeholders, including governments, businesses and non-governmental organizations, is essential to achieve SDGs. However, countries should focus their efforts. Our approach argues that, in the future, collaboration between economic, science and technology (S&T) and cultural fields needs to be strengthened.

The prosperity and stability of our future are inseparable from that of developing and least-developed countries [5]. However, within a single region, it is difficult to decouple economic growth from resource consumption. This suggests that economic collaboration between different countries is critically important in the transformation of the economic-growth model. Developed countries have made great advances in fields such as education, health care, the environment and big data; in certain underdeveloped regions, these advances can be used to resolve difficulties more effectively [3]. Sharing of these advances between different regions is conducive to improving our ability to cope with the various challenges on the SDGs.

In addition, culture, as the combination of knowledge, beliefs, institutions and customs, profoundly affects human behavior and is the key factor to determining whether sustainable development can be achieved [3]. Countries and regions comprise diverse cultures, thus resolving conflicts among different cultures is critical to achieving policy coherence promoting sustainable development [3]. Hence, on the basis of respecting cultural differences, we should work together to build a global sustainable culture to encourage joint action among all countries.

## CONCLUSION

The ultimate goal of sustainable development is to maintain the equilibrium and stability of our integrated socio-environmental system (ISES) [3]. However, if each country or agency focuses on an individual SDG in isolation, it could accelerate the collapse of the ISES. Hence, a systematic approach, which incorporates the characteristics of the ISES, can contribute to the synergy between different countries, agencies and stakeholders that will help to advance the overall realization of the SDGs.

Leaving no country behind is a major challenge in implementing the SDGs. The 3C approach introduced here combines the core ideas of recent relevant theoretical research and practices. To be successful, the 3C approach to achieving the SDGs requires a host of challenges to be overcome—the key one being achieving consensus. Considering that only 10 years remain before 2030, our study aims to provide a universal systematic approach to ensure the full implementation of the SDGs.
